# New Insights on Phytochemical Features and Biological Properties of *Alnus glutinosa* Stem Bark

**DOI:** 10.3390/plants11192499

**Published:** 2022-09-23

**Authors:** Antonella Smeriglio, Valeria D’Angelo, Anna Cacciola, Mariarosaria Ingegneri, Francesco Maria Raimondo, Domenico Trombetta, Maria Paola Germanò

**Affiliations:** 1 Department of Chemical, Biological, Pharmaceutical and Environmental Sciences (ChiBioFarAm), University of Messina, Viale Ferdinando Stagno d’Alcontres 31, 98166 Messina, Italy; 2 Fondazione “Prof. Antonio Imbesi”, Piazza Pugliatti 1, 98122 Messina, Italy; 3 PLANTA/Autonomous Center for Research, Documentation and Training, Via Serraglio Vecchio, 28, 90123 Palermo, Italy

**Keywords:** *Alnus glutinosa* (L.) Gaertn, black alder bark extract, diarylheptanoids, oregonin, antioxidant, anti-inflammatory, anti-angiogenic

## Abstract

*Alnus glutinosa* (namely black alder or European alder) is a tree of the Betulaceae family widely spread through Europe, Southeastern Asia, the Caucasus mountains, and Western Siberia. Its bark is traditionally used for medicinal purposes as an astringent, cathartic, febrifuge, emetic, hemostatic, and tonic, suggesting that it may contain bioactive compounds useful to counteract inflammation. The aim of this study was to investigate the phytochemical profile of *A. glutinosa* stem bark extract (AGE) by LC-DAD-ESI-MS/MS analysis and to validate some biological activities such as antioxidant, anti-inflammatory and anti-angiogenic properties by in vitro and in vivo models (chick chorioallantoic membranes and zebrafish embryos), that can justify its use against inflammatory-based diseases. The AGE showed a high total phenols content expressed as gallic acid equivalents (0.71 g GAE/g of AGE). Diarylheptanoids have been identified as the predominant compounds (0.65 g/g of AGE) with oregonin, which alone constitutes 74.67% of the AGE. The AGE showed a strong and concentration-dependent antioxidant (IC_50_ 0.15–12.21 µg/mL) and anti-inflammatory (IC_50_ 5.47–12.97 µg/mL) activity. Furthermore, it showed promising anti-angiogenic activity, inhibiting both the vessel growth (IC_50_ 23.39 µg/egg) and the release of an endogenous phosphatase alkaline enzyme (IC_50_ 44.24 µg/embryo). In conclusion, AGE is a promising source of antioxidant, anti-inflammatory and angio-modulator compounds.

## 1. Introduction

The genus *Alnus* (Betulaceae) includes more than 40 species, many of them with a long history of traditional use. *Alnus glutinosa* (L.) Gaertn, known as black alder or European alder, is a tree widespread in Europe and Asia. Many studies have shown that black alder bark extracts display antimicrobial, anti-inflammatory and anticancer activities [1]. However, in addition to being a promising plant matrix from a phytotherapeutic point of view, we must not forget that the bark of trees represents a forestry waste, which accounts for up to 12 % of the total wood-based biomass [2]. Considering this, a recent study has focused on the recovery of this important biomass to obtain high quality black alder bark extracts with a high content of total phenols and strong antioxidant activity, by using green chemistry techniques [2]. Phytochemical investigations have revealed that many bioactive compounds including phenolic acids, flavonoids, diarylheptanoids, terpenoids and steroids, were isolated from *A. glutinosa* stem bark [3]. Despite this, a very different chemical composition can be found among tree barks depending on growth place and pedo-climatic conditions [2]. 

Diarylheptanoids are polyphenols typically found in the genus *Alnus* and represent by far the most predominant bioactive compounds, although, to date, no quantitative phytochemical studies have been carried out. They consist of two aryl groups joined by a heptane chain in the main skeleton, with many substituents that can significantly influence their biological activity. Many diarylheptanoids have been reported to display antioxidant, antibacterial, antiviral, and anticancer activity [4]. Therefore, the recovery of this biomass certainly represents a very important source of bioactive compounds with promising health properties. 

It is well known that, in addition to the imbalance of the physiological antioxidant defense system and the onset of an acute inflammatory event, which often evolves into a chronic inflammatory state, an unregulated angiogenesis is also involved in many pathologies such as diabetic retinopathy, rheumatoid arthritis and other inflammatory-based diseases, also playing a pivotal role in tumor growth and metastasis formation [5]. Thus, nowadays there is a growing interest to discover new natural agents able to modulate these events for preventive and therapeutic purposes [6]. 

Considering this, the aim of the present study was to carry out, for the first time, a quali–quantitative characterization of *A. glutinosa* bark extract (AGE) and to investigate its antioxidant, anti-inflammatory and anti-angiogenic properties by several in vitro and in vivo models. Moreover, this study was designed also to investigate the role of the predominant diarylheptanoid found, oregonin, in the observed activities. 

## 2. Results

### 2.1. Phytochemical Profile

**Figure 1:**
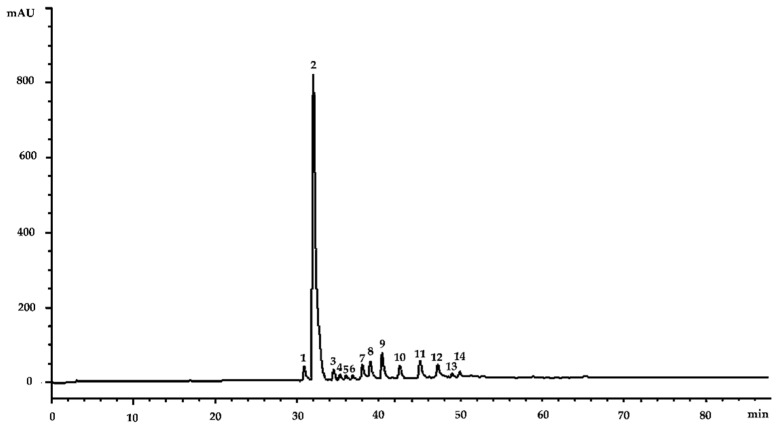
Representative LC-DAD chromatogram of *A. glutinosa* bark extract (AGE) acquired at 292 nm, a wavelength at which it is possible to observe all the identified compounds. Peak numbers, which follow the elution order, correspond to compounds listed in Table 1.

The extraction procedure adopted allowed the obtaining of a high extraction yield (24.36%). The dry extract (DE), suitably solubilized in methanol, was first subjected to a preliminary analysis for total phenols content with the Folin–Ciocalteu test, showing a high content of phenolic compounds expressed as gallic acid equivalents (0.71 g GAE/g DE). Subsequently, a qualitative characterization of AGE was carried out by means of LC-DAD-ESI-MS/MS. This analysis highlighted a very interesting phytochemical profile (Figure 1), showing a very high content of oregonin (Figure 1, peak no. 2), which alone represents about 74.50% of the whole extract. AGE is a very rich source of diarylheptanoids in general, with 12 other compounds identified belonging to the same class and representing about 25.20% of AGE. Moreover, two new diarylheptanoids were identified for the first time in black alder extract, hirsutanonol 5-*O*-glucoside and coumaroyl-oregonin. However, the extract appears poor in flavonoids (0.30%). The only compound identified belonging to this class of compounds is, in fact, a quercetin derivative, quercetin-3-sophoroside (Figure 1, peak no. 5). 

The quantitative analysis, obtained by expressing the content of diarylheptanoids as oregonin equivalents, and the content of quercetin-3-sophoroside as quercetin-3-*O*-glucoside equivalents, allowed us to highlight the most abundant compounds in AGE. The results were expressed as mean ± the standard deviation of three independent experiments in triplicate (*n* = 3) (Table 1). According to the quantitative analysis, oregonin is the predominant compound (484.18 mg/g DE), followed by hirsutenone (33.71 mg/g DE), rubranoside B (27.21 mg/g DE), 5 (S)-1.7-di (4-hydroxyphenyl)-5-*O*-β-D-[6-(E-3,4-dimethoxycinnamoylglucopyranosyl)] heptane-3-one (21.88 mg/g DE), 5-*O*-methylhirsutanonol (17.91 mg/g DE), 5 (S)-1-(4-hydroxyphenyl)-7-(3,4-dihydroxyphenyl)-5-*O*-β-D-[6-(3,4-dimethoxycinnamoylglucopyranosyl)] heptane-3-one (16.48 mg/g DE) and hirsutanonol-5-*O*-glucoside (13.95 mg/g DE).

### 2.2. Biological Properties

In this study, the antioxidant, anti-inflammatory and anti-angiogenic properties of AGE were evaluated. To this regard, both in vitro tests based on different environments and reaction mechanisms, and in vivo tests on two of the most accredited models for the study of angio-modulating substances, were carried out. 

As regards the antioxidant activity, six different tests were carried out aimed at evaluating: (i) the ability of the extract under examination to protect from heat-induced lipid peroxidation (β-carotene bleaching assay, BCB); (ii) the free radical scavenging activity against 1,1-diphenyl-2-picrylhydrazyl radical (DPPH); (iii) the ferric-reducing antioxidant power (FRAP); (iv) the trolox equivalent antioxidant capacity (TEAC); (v) the oxygen radical absorbance capacity (ORAC assay) and, finally, (vi) the iron-chelating activity (ferrozine assay).

**Figure 2:**
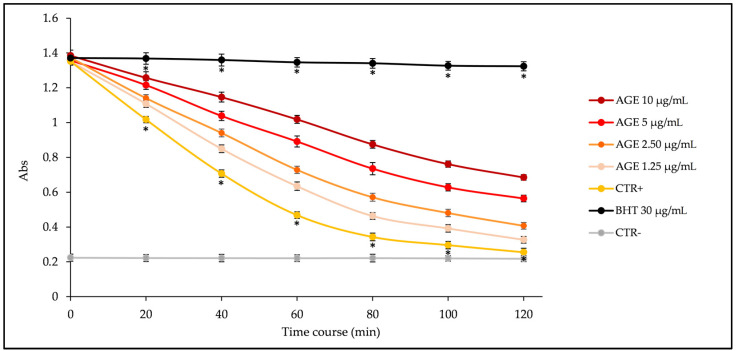
β-Carotene bleaching kinetic curves of *A. glutinosa* bark extract (AGE, 1.25–10 µg/mL) compared to the reference standard butylated hydroxytoluene (BHT) 30 µg/mL, positive control (CTR+), which consist in the same emulsion with methanol instead of sample and negative control (CTR-), which consist in the same emulsion without β-carotene and with methanol instead of sample. * *p* < 0.001 vs. AGE (1.25–10 µg/mL).

Figure 2 shows the kinetic curves (absorbance decrease vs. time expressed in minutes) of different concentrations of AGE (1.25–10 µg/mL) compared with the reference standard butylhydroxytoluene (BHT, 30 µg/mL), a synthetic antioxidant well known for its inhibiting properties on lipid peroxidation, and with the positive (β-carotene emulsion with the vehicle, i.e., methanol, instead of sample) and negative (blank emulsion without β-carotene) controls. As can be seen from Figure 2, AGE exhibits strong and concentration-dependent antioxidant activity, inhibiting, in a statistically significant manner (*p* < 0.001 vs. BHT and CTR+), the β-carotene bleaching in response to heat-induced linoleic acid peroxidation.

**Figure 3:**
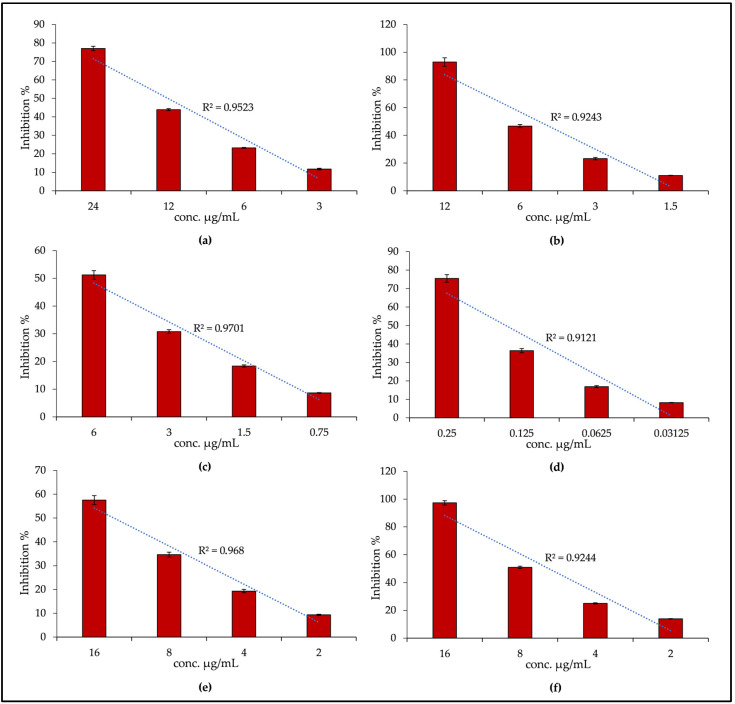
Antioxidant and anti-inflammatory activity of *A. glutinosa* stem bark extract (AGE): DPPH (**a**), FRAP (**b**), TEAC (**c**), ORAC (**d**), Bovine serum albumin (BSA) denaturation, (**e**) Protease inhibitory activity, (**f**) Panels (**a**–**e**) show the linear correlation coefficient (R^2^), which highlights the concentration-dependent behavior of AGE.

Figure 3 shows the results of DPPH (a), FRAP (b), TEAC (c) and ORAC assay (d) expressed as inhibition percentages (%). Despite AGE showing a strong antioxidant potency, it did not show any iron-chelating activity within the concentration range tested (50–400 µg/mL), well above the concentrations at which AGE showed promising antioxidant activity. As can be seen from Figure 3, the extract in this case also shows marked concentration-dependent antioxidant properties in all the tests carried out; indeed a calculated linear correlation index (R^2^) ≥ 0.9121 was observed. In any case, the AGE behavior is not always the same, and looking to the results obtained with respect to the AGE concentration tested, the following order of potency can be established: ORAC > TEAC > β-carotene bleaching > FRAP > DPPH.

In Figure 3, graph bars indicated with letters (e) and (f) refer to the anti-inflammatory activity, that was evaluated by two different in vitro assays: the bovine serum albumin (BSA) denaturation assay and the protease inhibitory activity. The first one evaluates the ability of AGE to counteract the heat-induced protein denaturation, whereas the second one evaluates the ability of AGE to inhibit one of the key enzymes involved in inflammatory-based diseases. 

In these two tests, AGE showed a quite comparable behavior, inhibiting both albumin denaturation in response to physical stress, and protease in a concentration-dependent manner (R^2^ ≥ 0.9244).

Given the promising antioxidant and anti-inflammatory properties of AGE and given the particular phytochemical profile of the extract, showing the oregonin as the predominant compound, the next step was to evaluate what was the contribution of this diarylheptanoid to the activities found. By expressing the results obtained as half-maximal inhibitory concentration (IC_50_) and calculating the respective 95% confidence limits (C.L.), it is possible to easily compare the results obtained with AGE, oregonin and the respective reference standards, as reported in Table 2.

Both AGE and oregonin showed very promising antioxidant and anti-inflammatory activities compared to the reference standards (RS). Specifically, trolox for DPPH, TEAC, FRAP and ORAC assay, BHT for β-carotene bleaching, and diclofenac sodium for anti-inflammatory tests were used as reference standards (Table 2). Interestingly, no statistically significant difference with respect to the RS was found for AGE in the DPPH, TEAC, FRAP, BSA denaturation assay and in terms of protease inhibitory activity, showing the extract an activity comparable with that of the respective RS. On the contrary, oregonin showed no statistically significant difference compared to the RS only in the following tests: TEAC, FRAP and protease inhibitory activity. However, even more interestingly, both the extract and the pure compound oregonin showed comparable antioxidant and anti-inflammatory activities, without any statistically significant difference in all the tests carried out, thus demonstrating that oregonin is the main compound responsible for the antioxidant and anti-inflammatory activities found in the present study.

Given these evidence and it being known that some diarylheptanoids have proved to be promising antitumor agents, the potential anti-angiogenic activity of AGE and oregonin using two in vivo models (chick chorioallantoic membranes and zebrafish embryos), was evaluated.

In the first case, the ability of AGE and the pure molecule oregonin to inhibit the growth of chick chorioallantoic membrane (CAM) blood vessels without causing toxicity was evaluated, whereas in the second case, their ability to counteract the release of the angiogenic marker endogenous alkaline phosphatase (EAP) from endothelial cells of zebrafish embryos, was investigated. In both cases, reference standards with proven anti-angiogenic activity were used: retinoic acid (CTR+, 1 µg/egg) and 2-methoxyestradiol (CTR+, 30 µg/embryo), respectively. 

**Figure 4:**
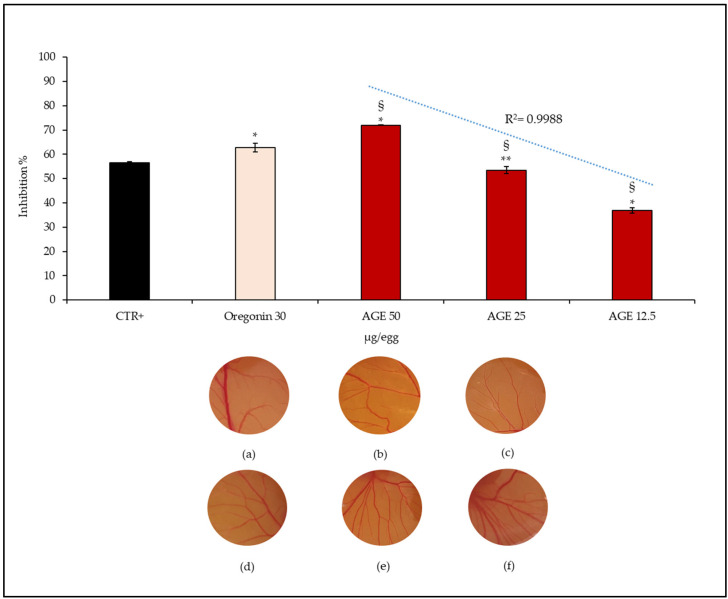
Anti-angiogenic properties of several concentrations of *A. glutinosa* bark extract (AGE) evaluated by CAM assay. In the figure, representative pictures of the CAMs after treatment with the positive control (retinoic acid, 1 μg/egg) (**a**), main bioactive compound of AGE at the same concentration present in the extract (oregonin, 30 μg/egg) (**b**), AGE 12.50–50 μg/egg (**c**–**e**) and negative control (deionized water) (**f**) were reported. The graph bar shows the linear correlation coefficient (R^2^), which highlights the concentration-dependent behavior of AGE. Results were expressed as mean inhibition percentage ± standard deviation of five independent experiments (*n* = 5). * < 0.001 vs. positive control (CTR+); ** < 0.05 vs. CTR+; ^§^ < 0.001 vs. oregonin 30 μg/egg.

**Figure 5:**
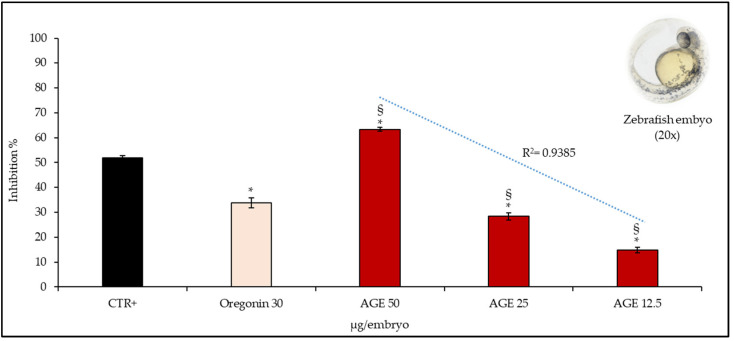
Anti-angiogenic properties of several concentrations of *A. glutinosa* bark extract (AGE, 12.5–50 μg/embryo) evaluated by EAP activity of vascular endothelial cells released from treated zebrafish embryos, of which a representative micrograph (magnification 20×) was reported. Results were expressed as mean inhibition percentage ± standard deviation of five independent experiments (*n* = 5). * *p* < 0.01 vs. positive control (CTR+), 2-methylestradiol 30 μg/embryo; ^§^ < 0.001 vs. oregonin 30 μg/embryo.3. Discussion.

Oregonin, as well as AGE, were tested at different concentrations, although in the graph bar shown in Figure 4 and Figure 5, it appears only at the maximum tested concentration (30 µg/egg and 30 µg/embryo, respectively), that it corresponds to the amount of oregonin within AGE at the highest concentration tested (AGE 50 µg/egg and 50 µg/embryo, respectively). 

As can be seen from Figure 4, AGE shows marked concentration-dependent anti-angiogenic properties (IC_50_, 23.39 µg/egg, C.L. 16.78–28.15), as can be deduced from the calculated linear correlation index (R^2^ = 0.9988) and as it possible to observe also from the pictures c–e (Figure 4). The highest concentration tested shows significantly higher anti-angiogenic activity (Figure 4c) than the CTR+ (Figure 4a). Conversely, the two lowest concentrations (Figure 4d,e) showed significantly lower anti-angiogenic activity (*p* < 0.05 and *p* < 0.001 for AGE 25 and 12.50 µg/mL, respectively) compared to the CTR+ (Figure 4a). Interestingly though that, in this case, the activity of the oregonin (IC_50_, 23.89 µg/egg, C.L. 14.22–34.88) is also significantly higher than the positive control (Figure 4b); however, at the same concentration present within the AGE 50 µg/egg (Figure 4c), the anti-angiogenic activity is significantly lower, suggesting the involvement of other bioactive compounds in the biological activity recorded.

This hypothesis was confirmed by the second in vivo model. Indeed, as can be seen from the graph bar in Figure 5, AGE inhibits the release of EAP from zebrafish embryos (IC_50_, 44.24 µg/embryo, C.L. 37.53–55.26) in a concentration-dependent manner (R^2^ ≥ 0.9385), with a superimposable behavior to that found in the previous in vivo model, with statistically significant results at all concentrations tested, both towards CTR+ and oregonin (IC_50_, 44.42 µg/embryo, C.L. 35.21–53.84). The latter, also in this case, contributes only partially to the activity of AGE (50 µg/embryo), confirming the hypothesis previously postulated. 

Finally, it should be noted that in the two in vivo models, at the effective concentrations tested, no toxicity was found, suggesting a potentially safe and effective use of both AGE and pure compound oregonin.

The extraction method adopted in this study allowed the obtaining of a stem bark extract with a high extraction yield (24.36% vs. 2.45–16.41%) and with a higher total phenol content (about five times greater) than those obtained with other extraction methods that used pure organic solvents or aqueous mixtures [2,7,8]. Similar results have been obtained instead, in terms of extraction yield and total phenols, with new green chemistry techniques, mainly using deionized water as an extraction solvent at high temperatures [2], although this latter irremediably determines a decrease of polyphenols, compounds well-known for their thermolability, giving greater relevance to terpenoids, steroids and tannins. Therefore, it is important, in addition to looking at the greener choice, also to identify the most suitable extraction technique to obtain the best results in terms of yield of the most interesting classes of bioactive compounds for specific health applications.

As can be seen from a recent review, which reports a complete picture of the chemical constituents of the genus *Alnus* [1], the extraction method adopted in the present study allowed us to obtain an extract rich in polyphenols at the expense of terpenoids. To date, 25 diarylheptanoids, 3 ellagitannins, 2 flavonoids and 13 terpenoids have been identified in *A. glutinosa*. Obviously, the phytochemical profile varies greatly depending on the part of the plant taken into consideration and depending on the type of extraction method adopted. However, of the 25 reported diarylheptanoids, 11 were identified in the present study, and only one of the two previously reported flavonoids, namely quercetin-3-sophoroside. In addition, two diarylheptanoids, a derivative of hirsutanol, hirsutanonol 5-*O*-glucoside, and the coumaroyl-derivative of oregonin, were identified for the first time in *A. glutinosa*. In addition, another certainly innovative aspect of the present study is to have carried out a qualitative and quantitative characterization of AGE. So far, the few studies available on *A. glutinosa* have focused on the isolation of the predominant diarylheptanoids, which have been identified according to their NMR, UV, IR, CD and mass spectra data [1]. The qualitative and quantitative characterization by LC-DAD-ESI-MS/MS has thus made it possible to obtain more information about the quantitative distribution of the various diarylheptanoids, giving a more rational perspective about the possible uses of AGE as a plant complex, as a rich source of diarylheptanoids and, in particular, of oregonin, other than establishing a more concrete structure–activity relationship of the contribution of each single constituent to the total activity. 

There are many reports that testify that extracts and isolated compounds from different *Alnus* species have significant antimicrobial, immunomodulatory, antioxidant and anti-inflammatory activity. Other than stem bark, other *A. glutinosa* parts investigated were buds and leaves, the first ones for their potential for healing upper airway diseases [9], and the second ones for their antimicrobial activity against some Gram-positive and Gram-negative bacteria, and *Candida albicans* [10]. Among other things, some commercial products based on buds or bud extracts of *A. glutinosa* are commercially available as food supplements and herbal remedies, respectively, for acute and chronic inflammatory processes affecting the mucous membranes.

According to our results, it has been reported that oregonin and hirsutenone, the most abundant compounds detected in this study, showed the strong ability to scavenge oxygen radicals compared with the positive controls in TEAC, DPPH and ORAC assay [11,12,13]. Furthermore, many other compounds also exhibited remarkable free radical scavenging capacity, e.g., rubranoside A [2]. Examining the structure/activity relationship, it has been demonstrated that hirsutenone, hirsutanonol, oregonin and rubranoside B, which possesses two 3,4-dihydroxyphenyl rings, were more active against ROS than alnuside A and alnuside B, which have a 3,4-dihydroxyphenyl ring and a 4-hydroxyphenyl ring. On the contrary, platyphylloside, which has two 4-hydroxyphenyl rings, showed weak activity [11], making it possible to affirm that the scavenging capacity against peroxyl radicals is closely related to the phenolic hydroxyls. This assumption was corroborated by some other studies, which also revealed that the phenolic hydroxyls were essential to the higher antioxidative activity of diarylheptanoids [14,15], with respect to other bioactive compounds. Finally, recently it has been observed that the catechol moiety was very important for the free radical scavenging property. Regarding the plant complex, this is the first study that evaluated the antioxidant and anti-inflammatory activity of a black alder bark extract with different in vitro tests based on different environments and reaction mechanisms. The only study available to date that has evaluated the antioxidant activity of *A. glutinosa* bark extracts is that of Lauberts and Pals [2]. They specifically evaluated and correlated the influence of different extraction techniques with the antioxidant activity found by DPPH, TEAC and ORAC tests, obtaining at best, comparable results to those obtained in the present study.

Regarding anti-inflammatory activity, Kim et al. [16], who isolated nine diarylheptanoids from the bark of *A. japonica*, showed that oregonin and hirsutenone exhibited the most promising inhibitory effects on lipopolysaccharide (LPS)-induced NO and COX-2 production [16], suggesting that the presence of a keto–enol group in the heptane moiety or a caffeoyl group in the aromatic ring was important for the inhibitory activity [16]. Subsequently, another study highlighted the importance of the carbonyl group for the efficacy of diarylheptanoids against LPS-induced NO production [17]. Moreover, Lee et al. [18] gave a possible molecular mechanism noting that oregonin was able to inhibit the inducible nitric oxide synthase (iNOS) gene transcription via suppressing transcriptional activity of nuclear factor kappa-light-chain-enhancer of activated B cells (NF-κB) and activator protein-1 (AP-1), as well as upregulating the anti-inflammatory molecule HO-1 [16]. In addition, oregonin was able to counteract lipid accumulation, inflammation, and ROS production in primary human macrophages [19]. Finally, hirsutenone may exert a preventive effect against LPS-induced inflammatory skin diseases through inhibition of extracellular signal-regulated kinase (ERK) pathway-mediated by NF-κB activation [20,21]. Rubranoside B and hirsutanolol isolated by the bark of *A. hirsuta*, and alnuside A, oregonin and platiphylloside isolated from *A. firma* showed significant ability to inhibit LPS-induced inflammation in macrophages or BV2 microglial cells [14,17]. Finally, Sajida et al. [22], evaluated the methanol extract and derived fractions of *A. nitida* stem bark for anti-inflammatory activity by using in vitro heat-induced albumin denaturation assay and various in vivo models, suggesting that diarylheptanoids can play a pivotal role in the anti-inflammatory and analgesic activities of the plant complex. 

All these evidence confirm what we observed in the present study, namely that the antioxidant and anti-inflammatory activity of AGE is mainly attributable to diarylheptanoids and that oregonin, which is the predominant compound in this extract, plays a pivotal role in the antioxidant and anti-inflammatory activity of the plant complex, although hirsutenone and other minor compounds may also contribute marginally.

Despite several studies investigating the antioxidant and anti-inflammatory activities of isolated diarylheptanoids, and some plant complexes belonging to several *Alnus* species, no data are today available about their potential anti-angiogenic activity. However, to investigate the molecular mechanisms underlying the anticancer effect of diarylheptanoids, recognized as the most important biological effect of these compounds, some studies have been conducted recently in order to evaluate the antiangiogenic properties of some isolated compounds. Hu et al. [23] found that the diarylheptanoid 1-phenyl-7-(4-hydroxy-3-methoxyphenyl)-4E-en-3-heptanone (PHMH) from *Alpinia officinarum* exerts anti-angiogenic activity both in vitro, ex vivo and in vivo. PHMH significantly inhibited vascular endothelial growth factor (VEGF)-induced vessel sprout formation ex vivo on the aorta ring, blood vessel formation in mice, angiogenesis in zebrafish, and newly formed blood vessels in the CAM model. These findings are in line with the inhibitory effects recorded on cell proliferation and migration observed in HUVECs. The authors hypothesized, as a possible molecular mechanism, the inhibition of VEGFR-2 auto-phosphorylation and the subsequent inhibition of the downstream targets protein kinase B (Akt)/mammalian target of rapamycin (mTOR), extracellular regulated kinase ½ (ERK1/2), and focal adhesion kinase (FAK). 

These evidences were corroborated by another recent study [24], which demonstrated that the diarylheptanoid (S,E)-1-(3,4 dimethoxyphenyl)-6-hydroxy-7-phenylhept-4-en-3-one (DPHP), an Alpinoid C analogue, suppressed the angiogenesis by inhibiting expression of factor-inhibiting hypoxia-inducible factor (HIF-1α), VEGF and its downstream signaling pathway, preventing also the vasculogenic process in the CAM model. Both studies used concentrations ranging from 0–20 µM that, considering the molecular weight of molecules, are comparable concentrations with respect to those tested in the present study for oregonin. 

Currently there are no studies available that investigate the possible structure/activity relationship concerning anti-angiogenic activity, being an almost unexplored field, but in the light of the previous observations concerning antioxidant and anti-inflammatory activity, it would be interesting to carry out the same type of studies with the diarylheptanoids typical of the genus *Alnus* and in particular with oregonin with reference to the *A. glutinosa* species, which together with hirsutenone has proved to be the most promising compound from the biological point of view.

## 3. Materials and Methods

### 3.1. Chemicals

The Folin–Ciocalteu reagent, gallic acid, sodium carbonate (Na_2_CO_3_), β-carotene, linoleic acid, Tween 40, chloroform, butylated hydroxytoluene (BHT),1,1-diphenyl-2-picrylhydrazyl (DPPH), 6-hydroxy-2,5,7,8-tetramethylchromane-2-carboxylic acid (trolox), 2,4,6-tris(2-pyridyl)-S-triazine (TPTZ), iron sulphate heptahydrate (FeSO_4_·7H_2_O), sodium acetate (CH_3_COONa), glacial acetic acid, 22,20-azino-bis(3-ethylbenzothiazoline-6-sulfonic acid (ABTS), potassium persulfate (K_2_S_2_O_8_), 2,2′-azobis(2-methylpropionamidine) dihydrochloride (AAPH), fluorescein sodium salt, sodium phosphate dibasic (Na_2_HPO_4_), potassium phosphate monobasic (KH_2_PO_4_), ferrozine, iron chloride tetrahydrate (FeCl_2_·4H_2_O), ethylenediaminetetraacetic acid (EDTA), bovine serum albumin (BSA) heatshock fraction protease, fatty acid and essentially globulin free (pH 7, ≥98%), trypsin from porcine pancreas Type IX-S lyophilized powder (13,000–20,000 BAEE units/mg protein), perchloric acid, trizma-base, casein, diclofenac sodium, retinoic acid, diethanolamine, p-nitrophenyl phosphate disodium salt and sodium hydroxide (NaOH) were purchased from Merck (Darmstadt, Germany). Acetonitrile, formic acid and oregonin (purity ≥ 95%) were HPLC grade and were purchased from Merck (Darmstadt, Germany). Quercetin-3-*O*-glucoside were HPLC grade (purity ≥ 99%) and was purchased by Extrasynthese (Geney, France). Where not specified, the reagents were of analytical grade.

### 3.2. Plant Material and Sample Preparation

*A. glutinosa* stem bark was collected from different trees (*n* = 5) on the Nebrodi (Messina, Italy) mountain ridge during April 2020. A voucher specimen (20/04 AG), identified by Prof. F. M. Raimondo, was deposited with the Department of Chemical, Biological, Pharmaceutical and Environmental Sciences, University of Messina (Italy). After collection, the bark was dried at room temperature (RT) until the moisture content was ≤10%, powdered (355 sieve) with a blade mill (IKA^®^ A11 basic analytical mill, IKA^®^-Werke GmbH & Co. KG, Staufen, Germany) and subsequently extracted according to the method reported by Smeriglio et al. [25] with some modifications. Briefly, 100 g of stem bark powder were extracted under stirring with 500 mL of methanol/water (80:20, *v*/*v*) mixture at room temperature for 72 h. Extraction was repeated three times, the supernatants recovered, filtered on Whatman paper no. 1, and dried by a rotary evaporator at 37 °C (Büchi R-205, Cornaredo, Italy) obtaining an extraction yield of 24.36%. The dried extract (AGE), with a nitrogen headspace, was stored at −20 °C until the subsequent analyses.

### 3.3. Total Phenols

Total phenols were quantified according to Cornara et al. [26]. Fifty microliters of sample solution (AGE) and gallic acid as reference compound, both at the same concentration range (75–600 μg/mL), were added to 500 μL of the Folin–Ciocalteu reagent and 450 μL of deionized water, incubating for 3 min. After this, 50 μL of 10% Na_2_CO_3_ *w*/*v* were added to the mixture, and samples were left in dark at RT for 60 min, mixing every 10 min. The absorbance was read at 785 nm against a blank consisting of methanol instead of sample by using an UV-Vis spectrophotometer (Shimadzu UV 1601, Kyoto, Japan). Results were expressed as g of gallic acid equivalents (GAE)/g of AGE.

### 3.4. Phytochemical Analysis

The phytochemical profile of AGE was investigated by liquid chromatography coupled with diode array detection and electrospray/ion-trap tandem mass spectrometry (LC-DAD-ESI-MS/MS) analysis. Separation was carried out by a Luna Omega PS C18 column (150 × 2.1 mm, 5 μm; Phenomenex, Torrance, CA, USA) at RT and with a flow rate of 0.4 mL/min using 0.1% formic acid (Solvent A) and methanol (solvent B) as mobile phase, according to the elution program and mass spectrometer parameters reported in Smeriglio et al. [27]. Mass spectra were acquired using a fragmentation energy of 1.2 V (MS/MS). The peaks were identified by comparing retention times, UV–VIS and mass spectra of the analytes with those of commercially available HPLC-grade reference standards (purity ≥ 95%), as well as by comparison with those reported in literature, and quantified by using external calibration curves of oregonin (1–50 μg/mL) for diarylheptanoids and quercetin-3-*O*-glucoside for quercetin derivatives. 

### 3.5. In Vitro Antioxidant and Anti-Inflammatory Activity

The antioxidant and anti-inflammatory activity of AGE was evaluated by several in vitro assays based on different mechanisms and reaction environments. The results, which represent the average of three independent experiments in triplicate (*n* = 3), were expressed as inhibition percentage (%) of the oxidative/radical/inflammatory activity, calculating the half-maximal inhibitory concentration (IC_50_) with the respective confidence limits at 95% (C.L.) by Litchfield and Wilcoxon’s test using PHARM/PCS software version 4 (MCS Consulting, Wynnewood, PA, USA). All the concentration ranges following the reported refer to the final concentrations of AGE and reference compounds in the reaction mixture. The spectroscopic and fluorometric measures were recorded by an UV-Vis spectrophotometer (Shimadzu UV 1601, Kyoto, Japan), an UV-VIS plate reader (Multiskan GO; Thermo Scientific, Waltham, MA, USA) and a fluorescence plate reader (Fluostar Omega, BMG Labtech, Ortenberg, Germany).

#### 3.5.1. BCB Assay

The BCB assay was carried out using a β-carotene emulsion prepared according to Muscarà et al. [28]. Briefly, 0.320 mL of AGE methanol solution (1.25–10 µg/mL), oregonin (2.5–20 μg/mL) and reference standard (BHT, 30 µg/mL) were added to 8 mL of β-carotene emulsion. An emulsion without β-carotene was used as a negative control, whereas a β-carotene emulsion with methanol instead of sample was used as positive control. The absorbance was recorded at starting time and during incubation at 50 °C, every 20 min for 120 min at 470 nm, recording the absorbance decay. 

#### 3.5.2. DPPH Assay

The DPPH assay was carried out according to Smeriglio et al. [29]. Briefly, 37.5 μL of AGE methanol solution (3.0–24.0 μg/mL), oregonin (2.5–20 μg/mL) and trolox as reference compound (1.25–10.0 μg/mL) were added to 1.5 mL fresh 10^–4^ M DPPH methanol solution, mixed and incubated in the dark for 20 min. The absorbance was recorded at 517 nm against a blank consisting of methanol instead of sample.

#### 3.5.3. FRAP Assay

The ferric-reducing antioxidant power was evaluated according to Denaro et al. [30]. Fifty microliters of AGE methanol solution (1.5–12 μg/mL) and oregonin (1.25–10 μg/mL) were added to 1.5 mL of fresh pre-warmed (37 °C) working FRAP reagent and were incubated for 4 min at RT in the dark. The absorbance was recorded at 593 nm by using trolox as reference compound (0.63–5.0 μg/mL) and methanol as blank.

#### 3.5.4. TEAC Assay

The radical scavenging activity was evaluated according to Danna et al. [31]. The reaction mixture (4.3 mM K_2_S_2_O_8_:1.7 mM ABTS, 1:5 *v*/*v*), was incubated for 12 h in the dark at RT, diluted to get an absorbance of 0.7 ± 0.02 (734 nm), and used within 4 h. Fifty microliters of AGE (0.75–6.0 μg/mL) and oregonin (1.25–10 μg/mL) were added to the reaction mixture (1 mL) and were incubated at RT for 6 min. The decrease in absorbance was recorded at 734 nm by using trolox as reference compound (0.63–5.0 μg/mL) and methanol as blank.

#### 3.5.5. ORAC Assay

The ORAC assay was carried out according to Bazzicalupo et al. [32]. Briefly, 20 µL of AGE methanol solution (0.031–0.25 μg/mL) and oregonin (0.03–0.20 μg/mL), were added to 120 μL of fresh 117 nM fluorescein and were incubated for 15 min at 37 °C. Sixty microliters of 40 mM of AAPH were added to trigger the reaction, which was recorded every 30 s for 90 min (λ_ex_ 485; λ_em_ 520) by the fluorescence plate reader reported in Section 3.5 and by using trolox as reference compound (0.25–2.5 μg/mL).

#### 3.5.6. Ferrozine Assay

The iron-chelating activity was evaluated according to Smeriglio et al. [33]. Fifty microliters of AGE (50–400 μg/mL) were added to 2 mM FeCl_2_·4 H_2_O (25 µL) and were incubated at RT for 5 min. Fifty microliters of 5 mM ferrozine and 1375 µL of deionized water were then added to the reaction mixture. The absorbance was recorded after 10 min at 562 nm. EDTA was used as reference compound (1.25–10 μg/mL).

#### 3.5.7. BSA Denaturation Assay

The ability of AGE to inhibit the heat-induced BSA denaturation was evaluated according to Smeriglio et al. [34]. Briefly, 100 µL of 0.4 % BSA solution and 20 µL of PBS (pH 5.3) were added into a 96-well plate. Therefore, 80 µL of AGE methanol solution (2.0–16.0 μg/mL) and oregonin (1.25–10.0 μg/mL), were added to the mixture. The absorbance was recorded immediately at 595 nm and after incubation (30 min at 70 °C). A blank consisting of methanol was used as blank. Diclofenac sodium was used as a reference compound (3.0–24.0 μg/mL).

#### 3.5.8. Protease Inhibition Assay

The protease inhibitory activity of AGE was evaluated according to Smeriglio et al. [35]. Briefly, 200 µL of AGE methanol solution (2.0–16.0 μg/mL) and oregonin (1.25–10.0 μg/mL) were added to the reaction mixture consisting of 10 μg/mL trypsin (12 μL) and 25 mM Tris-HCl buffer pH 7.5 (188 μL). Two-hundred microliters of 0.8% casein was added and the reaction mixture incubated for 20 min at 37 °C in a water bath. After this, 400 μL of perchloric acid was added to stop the reaction. The cloudy suspension was centrifuged at 3500× *g* for 10 min and the absorbance of the supernatant was recorded at 280 nm against a blank consisting of methanol. Diclofenac sodium was used as a reference compound (2.0–16.0 μg/mL).

### 3.6. In Vivo Anti-Angiogenic Activity

#### 3.6.1. CAM Assay

The evaluation of the anti-angiogenic properties of AGE was carried out according to Smeriglio et al. [36]. Fertilized eggs of *Gallus gallus* were incubated for 96 hrs at 37 °C to promote the vessels’ growth and the CAM development. Malformed or dead embryos were discarded. At first, a screening to find a non-toxic concentration range for AGE and oregonin, was carried out. After this, AGE methanol solution (12.50–50 μg/egg), oregonin (3.75–30 μg/egg), retinoic acid (1 μg/egg) as positive control or methanol as negative control were applied on the CAM surfaces and the eggs were incubated for 24 h. The anti-angiogenic effect was evaluated by a stereomicroscope (SMZ-171 Series, Motic, Hong Kong, China) equipped with a digital camera (Moticam^®^ 5 plus). Micrographs were processed by GNU Image Manipulation Program (GIMP version 2.10.2). Results, obtained by three independent experiments in quintuplicate (*n* = 5) were expressed as reported in Section 3.5.

#### 3.6.2. EAP Activity on Zebrafish Embryos

The quantification of EAP in zebrafish embryos was carried out according to Certo et al. [37] following the ethical guidelines described by the National Institute of Health Guide for Care and Use of Laboratory Animals. Male and female zebrafish specimens were purchased from a local pet store and kept in an aquarium at 28 °C with a 14/10 (light/dark) photoperiod. After a natural mating, embryos were collected and kept in the water at 28.5 °C. Twenty-four hours post-fertilization (hpf), the healthy and regular embryos were selected. At first, wider concentration ranges were tested to check possible toxicity of AGE and oregonin.

After dechorionizing, embryos were seeded in a 96-well plate and incubated with 100 μL of embryos water containing AGE methanol solution (12.50–50 μg/embryo), oregonin (3.75–30 μg/embryo) and reference standard 2-methoxyestradiol (30 μg/embryo). Methanol was used as negative control. All embryos were incubated for 72 hpf. After that, embryos were dehydrated by increasing concentrations of ethanol, washed three times with 1M diethanolamine buffer (pH 9.8) and then incubated for 30 min at RT with p-nitrophenyl phosphate disodium salt. Sodium hydroxide (2 mM) was added to stop the reaction. The absorbance was recorded at 405 nm by the microplate reader reported in Section 3.5. Results, obtained by three independent experiments in quintuplicate (*n* = 5), were expressed as reported in Section 3.5.

### 3.7. Statistical Analysis

Results were expressed as mean inhibition percentage, calculating the IC_50_ and respective C.L. at 95% of three independent experiments in triplicate (*n* = 3) for in vitro experiments and of three independent experiments in quintuplicate (*n* = 5) for in vivo experiments. The statistical significance was evaluated by one-way analysis of variance (ANOVA) followed by Tukey’s test, using SigmaPlot 12.0 software. Data were considered statistically significant for *p* < 0.05.

## 4. Conclusions

In conclusion, the extraction method adopted in the present study made it possible to obtain an extract of black alder bark very rich in diarylheptanoids. Two diarylheptanoids, hirsutanonol 5-*O*-glucoside and coumaroyl-oregonin, were identified for the first time. This is the first study to carry out a qualitative and quantitative characterization of black alder bark extract, identifying oregonin as the predominant compound followed by hirsutenone, two of the diarylheptanoids recognized, due to SAR studies, as capable of exerting the strongest antioxidant and anti-inflammatory activities. Indeed, the present study demonstrates, thanks to a direct comparison of the biological properties investigated between the plant complex and the most abundant diarylheptanoid oregonin, that the promising antioxidant, anti-inflammatory and anti-angiogenic activity found in the plant complex is almost entirely attributable to oregonin.

Although further studies are needed to investigate the molecular mechanisms underlying these interesting biological activities, the stem bark extract of *A. glutinosa* has certainly proved to be a very interesting and promising plant complex that could have applications in the cosmeceutical, nutraceutical, and pharmaceutical fields.

## Figures and Tables

**Table 1:** Qualitative and quantitative analysis of *A. glutinosa* stem bark extract (AGE) by LC-DAD-ESI-MS/MS analysis. Results, which are the mean ± standard deviation (S.D.) of three independent experiments in triplicate (*n* = 3), were expressed as mg of oregonin equivalents (OE)/g of dry extract (DE) for diarylheptanoids, and as mg of quercetin-3-*O*-glucoside equivalents (QGE)/g of DE for quercetin derivatives.

# ^a^	Compound	RT ^b^	[M-H]^−^	MS/MS	λ_max_	AGE
		(min)	(*m*/*z*)	(*m*/*z*)	(nm)	mg/g DE
1	Hirsutanonol 5-*O*-glucoside	31.01	507	345	232; 282	13.95 ± 0.02
2	Oregonin	32.23	477	327	234; 282	484.18 ± 1.52
3	Alnuside	34.42	461	311	228; 254	5.02 ± 0.05
4	Platyphylloside	35.18	475	189	228; 248	3.36 ± 0.02
5	Quercetin-3-sophoroside	36.14	625	464	280; 330	1.66 ± 0.01
6	Rubranoside A	37.05	493	331	228; 282	3.76 ± 0.01
7	5-*O*-Methylhirsutanonol	38.44	359	345	230; 282	17.91 ± 0.03
8	Rubranoside B	39.21	463	331	228; 282	27.21 ± 0.12
9	Hirsutenone	40.62	327	205	228; 282	33.71 ± 0.17
10	Coumaroyl-oregonin	42.63	623	477	222; 290	12.11 ± 0.05
11	5(*S*)-1,7-di(4-hydroxyphenyl)-5-*O*-β-D-[6-(*E*-3,4-DMC ^c^-glucopyranosyl)] heptane-3-one	45.44	665	475	223; 287	21.88 ± 0.08
12	5(*S*)-1-(4-hydroxyphenyl)-7-(3,4-dihydroxyphenyl)-5-O-β-D-[6-(3,4-DMC ^c^-glucopyranosyl)] heptane-3-one	47.53	681	475	223; 286	16.48 ± 0.14
13	3(*R*)-1,7-di(3,4-dihydroxyphenyl)-5-*O*-β-D-[6-(*Z*-3,4-DMC ^c^-glucopyranosyl)] heptane	49.42	683	493	224; 288	3.28 ± 0.03
14	3(*R*)-1,7-di(3,4-dihydroxyphenyl)-5-*O*-β-D-[6-(*E*-3,4,5-TMC ^d^-glucopyranosyl)] heptane	50.02	713	523	224; 289	3.61 ± 0.01
Diarylheptanoids						647.96
Oregonin						484.18
Flavonoids						1.66

# ^a^ = elution order; RT ^b^ = retention time; DMC ^c^, dimethoxycinnamoyl; TMC ^d^, trimethoxycinnamoyl.

**Table 2:** Determination of antioxidant and anti-inflammatory activity of *A. glutinosa* stem bark extract (AGE) by several in vitro assays based on different environments and reaction mechanisms. Results, which were expressed as half-maximal inhibitory concentration (IC_50_, µg/mL) with confidence limits at 95% (C.L.), are the mean of three independent experiments in triplicate (*n* = 3) and are compared to the main bioactive compound oregonin and the reference standard (RS).

Assay	AGE	Oregonin	RS ^b^
	IC_50_ (C.L. 95%) µg/mL		
BCB	8.95 (6.20–12.92) *	9.50 (7.07–11.44) *	0.24 (0.19–0.30)
DPPH	12.21 (10.26–14.52)	16.13 (13.85–18.67) *	8.35 (2.07–13.66)
TEAC	5.97 (4.69–7.60)	6.46 (4.08–8.62)	4.77 (3.85–5.90)
FRAP	4.75 (1.89–7.95)	7.01 (5.27–9.86)	5.27 (4.09–6.79)
ORAC	0.15 (0.12–0.17) *	0.17 (0.12–0.20) *	0.69 (0.31–1.53)
BSA ^a^ denaturation	12.97 (10.40–16.18)	9.35 (6.27–12.09) *	17.82 (14.15–22.44)
Protease inhibitory activity	5.47 (1.75–7.02)	6.38 (4.18–8.77)	6.33 (3.07–13.08)

^a^ BSA, Bovine serum albumin; ^b^ RS, reference standard, which is Trolox for DPPH, TEAC, FRAP and ORAC assay, butylated hydroxytoluene (BHT) for β-carotene bleaching assay and diclofenac sodium for anti-inflammatory assays; * *p* < 0.001 vs. RS.

## Data Availability

Not applicable.
